# Exploring Transcriptional Regulation of Hyperaccumulation in *Sedum plumbizincicola* through Integrated Transcriptome Analysis and CRISPR/Cas9 Technology

**DOI:** 10.3390/ijms241411845

**Published:** 2023-07-24

**Authors:** Yixin Zhang, Yanlan Mo, Liyuan Han, Zhenyuan Sun, Wenzhong Xu

**Affiliations:** 1 State Key Laboratory of Tree Genetics and Breeding, Key Laboratory of Tree Breeding and Cultivation of State Forestry Administration, Research Institute of Forestry, Chinese Academy of Forestry, Beijing 100091, China; zhangyixin7863@163.com (Y.Z.);; 2 Key Laboratory of Plant Resources, Institute of Botany, Chinese Academy of Sciences, Beijing 100093, China; 3 Key Laboratory of Soil Environment and Pollution Remediation, Institute of Soil Science, Chinese Academy of Sciences, Nanjing 210008, China; 4 University of Chinese Academy of Sciences, Beijing 100049, China; 5 China National Botanical Garden, Beijing 100093, China

**Keywords:** cadmium, CRISPR/Cas9, hyperaccumulation, transcription factor, *Sedum plumbizincicola*, transcriptome sequencing

## Abstract

The cadmium hyperaccumulator *Sedum plumbizincicola* has remarkable abilities for cadmium (Cd) transport, accumulation and detoxification, but the transcriptional regulation mechanisms responsible for its Cd hyperaccumulation remain unknown. To address this knowledge gap, we conducted a comparative transcriptome study between *S. plumbizincicola* and the non-hyperaccumulating ecotype (NHE) of *Sedum alfredii* with or without Cd treatment. Our results revealed many differentially expressed genes involved in heavy metal transport and detoxification that were abundantly expressed in *S. plumbizincicola*. Additionally, we identified a large number of differentially expressed transcription factor genes, highlighting the complexity of transcriptional regulatory networks. We further screened four transcription factor genes that were highly expressed in the roots of *S. plumbizincicola* as candidate genes for creating CRISPR/Cas9 knockout mutations. Among these, the *SpARR11* and *SpMYB84* mutant lines exhibited decreased Cd accumulation in their aboveground parts, suggesting that these two transcription factors may play a role in the regulation of the Cd hyperaccumulation in *S. plumbizincicola*. Although further research will be required to determine the precise targeted genes of these transcription factors, combined transcriptome analysis and CRISPR/Cas9 technology provides unprecedented opportunities for identifying transcription factors related to Cd hyperaccumulation and contributes to the understanding of the transcriptional regulation mechanism of hyperaccumulation in *S. plumbizincicola*.

## 1. Introduction

Cadmium (Cd) is a non-essential heavy metal element that is highly toxic to animals and most plants, and it has a long biological half-life of 25–30 years [1]. Cd contamination in the human body, even at trace levels through food chains, can cause serious diseases. However, a rare class of plants known as Cd hyperaccumulators have the unique ability to accumulate Cd above 0.01% of their dry weight or more than 100 mg/kg in their aerial parts under natural habitat conditions [2]. The effective uptake and accumulation of Cd by these hyperaccumulator plants make them suitable for phytoremediation, which involves cleaning up Cd-contaminated sites.

In hyperaccumulators, the functional genes responsible for heavy metal ion transport, chelation and sequestration are constitutively expressed at high levels in specific tissue to facilitate heavy metal hyperaccumulation and detoxification [3], which is contrary to the low and heavy metal-inducible expression in non-accumulator plants. For instance, the expression of zinc (Zn) root-to-shoot transporter gene *AhHMA4* was constitutive and significantly higher in hyperaccumulator *Arabidopsis halleri* than the homologous genes (*AtHMA2/AtHMA4*) found in *Arabidopsis thaliana* [4,5]. Furthermore, when comparing the transcriptome of the two ecotypes of *Noccaea caerulescens* (Ganges and Prayon) with contrasting Cd-accumulated ability, many genes encoding metal transporters were also more highly expressed in the Cd hyperaccumulator ecotype Ganges [6]. The Ganges ecotype of *N. caerulescens* exhibited seven times more expression of *NcHMA3* (heavy metal ATPase 3) than the non-accumulator ecotype Prayon [7]. Additionally, genes related to detoxification and lignin biosynthesis were also highly expressed in *N. caerulescens* but lowly expressed in *A. thaliana* [8].

As a strong Cd/Zn hyperaccumulator, *Sedum plumbizincicola* has also been found to exhibit high levels of expression of genes related to cadmium transport and detoxification [9]. Furthermore, cell wall synthesis-related genes were highly expressed in *S. plumbizincicola* [9]. SpHMA3, with its high-level constitutive expression, plays a critical role in the vacuolar sequestration and detoxification of Cd in young leaves and stems of *S. plumbizincicola* [10]. Additionally, SpHMA1 acts as a chloroplast Cd exporter and is also constitutively expressed in *S. plumbizincicola* with 100 times higher levels of *SaHMA1n* expression in the non-hyperaccumulating ecotype of *Sedum alfredii* [11]. Furthermore, *S. plumbizincicola* possesses a large biomass, fast growth rate and high resistance to cadmium compared to other hyperaccumulator plants, making it suitable for the remediation of contaminated soil [12].

Transcription factors are critical regulators of gene expression that control heavy metal uptake, accumulation and detoxification. Some transcription factors regulating Cd transport and tolerance have been isolated from non-accumulator plants, such as Arabidopsis, tobacco and rice. For example, AtMYB4 enhances the expression of *PCS1* and *MT1C* to increase the synergistic activity of the antioxidant defense system for protection against oxidative damage, thus regulating cadmium tolerance in *Arabidopsis thaliana* [13]. AtWRKY13 activates *PDR8* (an ABC transporter as a Cd extrusion pump conferring Cd tolerance) transcription by directly binding to its promoter, thereby positively regulating Cd tolerance in Arabidopsis [14]. Similarly, OsMYB45 has been shown to improve cadmium tolerance in rice by regulating the transcriptional expression of the catalase (CAT) gene [15]. These studies highlight the key role played by transcription factors in the transcription and expression of cadmium transporter or chelator genes. However, the transcription factors and molecular regulatory networks involved in the constitutively and highly expressed genes related to Cd hyperaccumulation are not well studied in hyperaccumulator plants.

With the development of gene sequencing technology, especially high-throughput transcriptome and whole-genome sequencing, researchers have isolated numerous transcription factors from various plant species. However, the biological functions of these transcription factors need to be further experimentally verified, and the most effective way is to obtain loss-of-function mutants of these genes through targeted gene mutations [16,17]. Progress in functional genomic research was long limited until 2013 when CRISPR/Cas genome-editing technology was applied in plants [18,19]. The CRISPR/Cas technology uses target-specific RNA to bring Cas nucleases to specific sites of target genes in the genome to cut target genes, resulting in mutations [20]. In order to explore the key transcription factors in the regulation of Cd tolerance and hyperaccumulation, we performed comparative transcriptome analysis between the hyperaccumulator *S. plumbizincicola* and the non-accumulator *S. alfredii* in response to Cd treatments. The expression profiles of differentially expressed genes revealed that Cd transport/detoxification-related genes were constitutively expressed at high levels in *S. plumbizincicola*, and some transcriptional regulatory genes also showed differential expression. Using CRISPR/Cas9 editing technology, we generated knockout mutants of four differentially expressed transcription factors and found that, after loss-of-function mutation, two of them changed the Cd accumulation traits in *S. plumbizincicola*. This study provides us a unique opportunity to discover Cd hyperaccumulation-related transcription regulatory genes in hyperaccumulator plants.

## 2. Results

### 2.1. Cd Accumulation Differences between S. plumbizincicola and S. alfredii (NHE)

**Figure 1:**
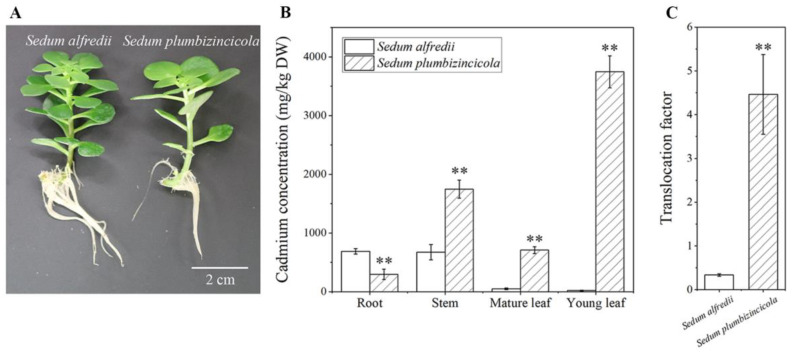
Plant morphology and cadmium (Cd) accumulation in two Sedum species exposed to 50 µM Cd for 10 days (**A**–**C**). Plant morphology (**A**). Cd concentrations in the root, stem, mature leaf and young leaf (**B**). Cd translocation factors (**C**). Note: bars refer to the mean ± standard error (SE) of four replicates. ** indicates highly significant differences between groups at 0.01 level (unpaired *t* test).

Although there were no obvious morphological differences between *S. plumbizincicola* and *S. alfredii* (NHE) rooted cuttings after a 10-day exposure to 50 µM Cd, there was a clear difference in Cd accumulation in the plants. Under 50 µM Cd treatment, the cadmium concentrations in the above-ground parts (stem, mature leaf and young leaf) of *S. plumbizincicola* were significantly higher than those in *S. alfredii* (NHE), especially in young leaves (Figure 1). The average concentration of Cd in the young leaves of *S. alfredii* (NHE) and *S. plumbizincicola* was 20.22 mg·kg^−1^ and 3747.06 mg·kg^−1^, respectively (Table S3). However, the Cd concentration in the roots of *S. alfredii* (NHE) was higher than that in the roots of *S. plumbizincicola*. It is worth mentioning that the translocation factor of Cd (the ratio of the Cd content in the shoot to that in the root) was 4.46 in *S. plumbizincicola* and 0.34 in *S. alfredii* (NHE), indicating a significant difference (Table S4).

### 2.2. RNA-Seq Analysis on S. plumbizincicola and S. alfredii under Cadmium Treatment

To investigate the molecular mechanisms underlying the differential Cd accumulation in the two Sedum species described above, we conducted RNA-Seq analyses to generate transcriptome profiles. After subjecting the plants to a 10-day exposure of 50 µM Cd, we obtained stable responses to cadmium and proceeded with sequencing. Following RNA-seq and data filtering, the raw reads ranged from 46650672 to 51495896 in each sample. The GC content was no less than 44.66%, and the Q20 and Q30 values were both above 97.67% and 93.02%, respectively (Table S5). Through sample correlation analysis, we identified and excluded one abnormal sample, leaving a total of 23 samples for subsequent analysis (Figure S1). As *S. plumbizincicola* and *S. alfredii* (NHE) belong to closely related species within the same genus, we mapped the second-generation sequences of both species to the *S. plumbizincicola* full-length transcriptome. Homologous genes of the two species were placed in the same number, which was conducive to subsequent analysis. For sequences that did not map, gene numbers were generated independently. The clean reads of each sample were mapped to the Sedum full-length reference transcription database obtained by our laboratory, and the comparison rate ranged between 64.13 and 91.19% (Table S6). By integrating full-length and next-generation transcriptome data, we obtained a total of 81693 transcripts. In order to confirm the sequencing quality, eight candidate genes that demonstrated high expression in *S. plumbizincicola* and were potentially associated with Cd accumulation were selected for qRT-PCR analysis. The results of relative expression were consistent with the sequencing data, indicating species and tissue-specific expression patterns (Figure S2).

### 2.3. Expression of Transcriptional Regulatory Genes

**Figure 2:**
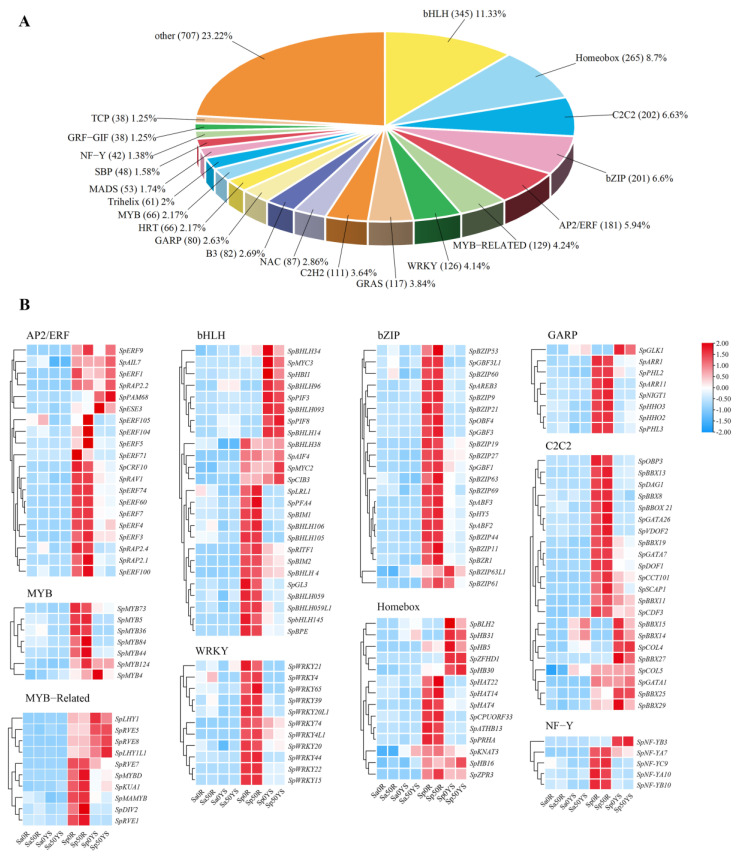
Transcription factor analysis. (**A**) Composition of transcription factor families. (**B**) Heat maps of some highly expressed transcription factor families. Note: in the heat map, average FPKM > 20 in the root or leaf of *S. plumbizincicola*. R: Roots, YS: Young shoots, Sa: *S. alfredii*, Sp: *S. plumbizincicola*.

We observed significant differences in the expression levels of some heavy metal-related genes between *S. plumbizincicola* and *S. alfredii* (NHE). Possibly, functional genes involved in heavy metal transport and detoxification were constitutively expressed at high abundance levels in *S. plumbizincicola* (Table S7). Transcription factors play important roles in regulating the differential expression of genes. To explore hyperaccumulation-related transcriptional regulatory genes in *S. plumbizincicola*, we used MapMan to retrieve a total of 3045 transcriptional regulatory genes in *S. plumbizincicola* and *S. alfredii* (NHE) without cadmium treatment and compared them (Table S8). These 3045 transcription factors belong to gene families such as bHLH, Homeobox, C2C2, bZIP, AP2/ERF, WRKY and others (Figure 2a). Using FDR < 0.05 and |log2FC| > 1 as thresholds, we identified 1904 differentially expressed transcription factors in the roots and 1788 differentially expressed transcription factors in the young shoots between *S. plumbizincicola* and *S. alfredii* (NHE) (Table S9). Additionally, we found that 43 and 137 differentially expressed transcription factor genes were induced in the roots and young shoots of *S. plumbizincicola*, respectively, after Cd exposure (Table S9). We analyzed the basic information and sequences of highly expressed transcription factors in *S. plumbizincicola* (genes with an average expression of FPKM > 20 in the roots or leaves); partial results are shown in Table 1, and all data are shown in the supplementary Table S10. Heat mapping of some families of highly expressed genes showed that most transcription factor genes highly expressed in *S. plumbizincicola* were constitutively expressed without cadmium induction. However, a small number of transcription factors highly expressed in *S. plumbizincicola* were cadmium-induced in *S. alfredii* (NHE) (Figure 2).

**Figure 3:**
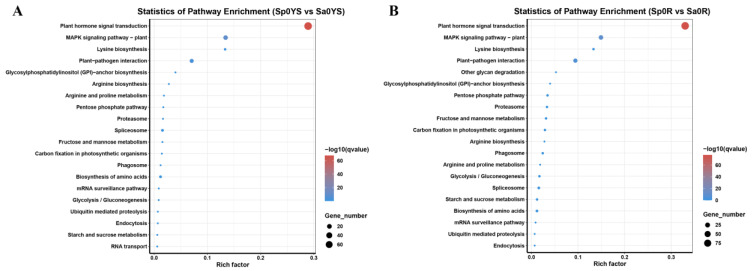
KEGG functional enrichment of differentially expressed transcription factor genes in *S. plumbizincicola* and *S. alfredii* (NHE) (**A**,**B**). Enriched KEGG pathways of Sp0YS vs. Sa0YS (**A**); enriched KEGG pathways of Sp0R vs. Sa0R (**B**). Note: R: Roots, YS: Young shoots, Sa: *S. alfredii* (NHE), Sp: *S. plumbizincicola*.

The KEGG enrichment scores indicated that the difference in transcription factor genes between *S. plumbizincicola* and *S. alfredii* (NHE) was mainly related to plant hormone signal transduction and MAPK signaling pathways (Figure 3). The plant hormone signal transduction pathways involved in ethylene include abscisic acid, salicylic acid, jasmonic acid, gibberellin, ethylene and auxin.

### 2.4. Creating Knockout Mutants of Four Transcription Factor Genes Using CRISPR/Cas9 System

**Figure 4:**
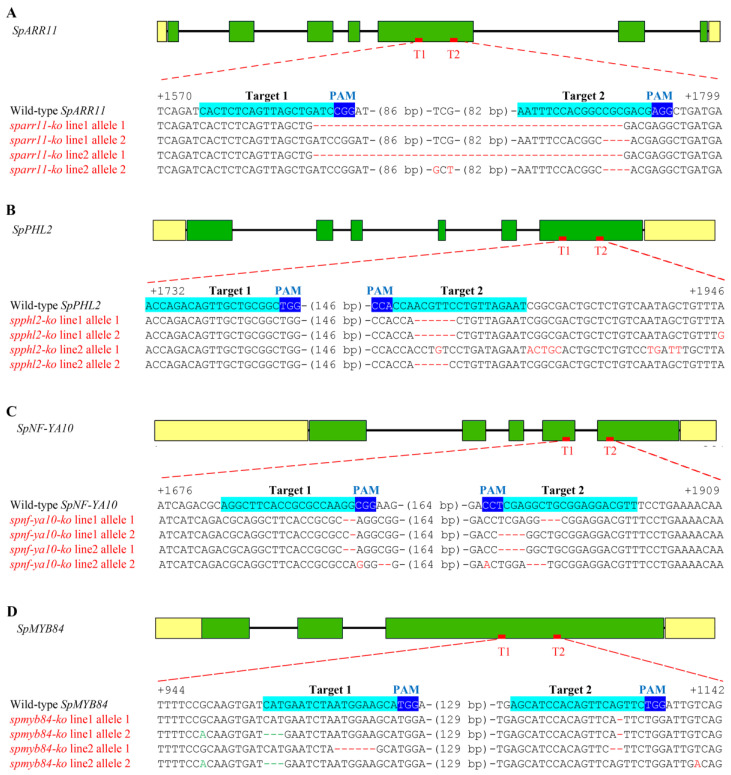
Genomic structure, target sites for CRISPR/ Cas9-engineered and sequences of *sparr11-ko* line1, 2 (**A**), *spphl2-ko* line1, 2 (**B**), *spnf-ya10-ko* line1, 2 (**C**) and *spmyb84-ko* line1, 2 (**D**) from independent first-generation transgenic plants. Note: In wild-type transcription factor genes, sgRNA targets are shaded in cyan, and protospacer-adjacent motif (PAM) sequences are shaded in blue. In knockout transcription factor genes, deleted nucleotides are replaced by red dashes, inserted or substituted nucleotides are indicated by red letters, and sequence gap length is indicated in parentheses.

To investigate whether transcription factors play a role in the hyperaccumulation of cadmium, we selected four highly expressed genes from the differentially expressed transcription factors in the roots of *S. plumbizincicola*, namely *SpARR11* (Sp-R_1-10k_transcript/35867), *SpPHL2* (Sp-R_1-10k_transcript/38907)*, SpNF-YA10* (Sp-R_1-10k_transcript/38848) and *SpMYB84* (Sp-R_1-10k_transcript/38968), for generating knockout mutants. Two target sites of each gene were designed based on the genomic sequences of their open reading frames (ORFs) (Figure 4). The sgRNAs were cloned into the CRISPR/Cas9 T-DNA vector pHSE401 and transformed into *S. plumbizincicola* plants via *Agrobacterium*-mediated transformation. We amplified the putative edited region of each targeted gene using gene-specific primers from all the positive transgenic lines and wild-type plants. We identified two independent knockout mutant lines for each target gene by sequencing the PCR products and selected them for subsequent experiments (Figure 4).

The *sparr11-ko* line1 plants had deletions in exon 5 of *SpARR11* resulting in a premature stop codon and deletions of 129 aa and 85 aa at the C-terminus of SpARR11. Similarly, the *sparr11-ko* line2 plants had a deletion and a substitution in the *SpARR11* genome leading to a premature stop codon and deletions of 129 aa and 86 aa at the C-terminus of SpARR11 (Figure 4A and Figure S3). *SpPHL2* encoded a 190 aa protein, while *spphl2-ko* line1 encoded a protein of 189 aa due to six base deletions in *SpPHL2*. By contrast, the *spphl2-ko* line2 plants had a deletion and a substitution in *SpPHL2* resulting in the removal of 27 aa and 43 aa at the C-terminus of SpPHL2 (Figure 4B and Figure S3). *SpNF-YA10* encoded a 210 aa protein, but *spnf-ya10-ko* line1 encoded proteins of 131 aa and 122 aa due to the removal of 79 aa and 88 aa, respectively, at the C-terminus of SpNF-YA10. The *spnf-ya10-ko* line2 plants had two deletions in *SpNF-YA10* leading to a premature stop codon and deletion of 79 aa at the C-terminus of *SpNF-YA10* and removal of 3 aa at the C-terminus (Figure 4C and Figure S3). *SpMYB84* encoded 237 aa and 234 aa proteins, *spmyb84-ko* line1 encoded 181 aa and 179 aa proteins due to deletions at the C-terminus, and *spmyb84-ko* line2 encoded 175 aa and 234 aa proteins (Figure 4D and Figure S3).

### 2.5. Cd Accumulation Analysis of the Transcription Factor Genes Knockout Mutants

**Figure 5:**
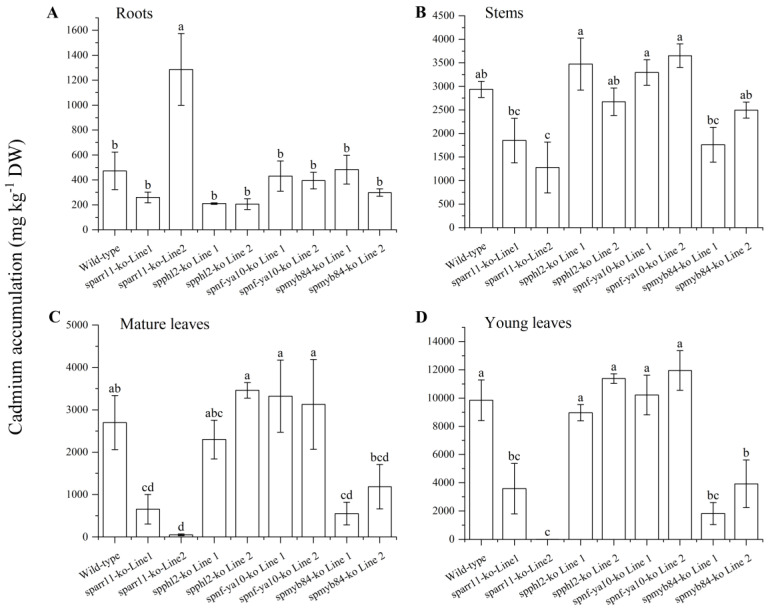
Cd accumulation was measured in the roots (**A**), stems (**B**), mature leaves (**C**) and young leaves (**D**) of both wild-type and knockout mutant lines of *S. plumbizincicola* under CdCl_2_ treatments. The plants were grown with 1/4 MS solution medium supplemented with 100 μM CdCl_2_ for ten days. Note: The bars represent the mean ± standard error (SE) of three replicates, and different letters indicate significant differences between groups at 0.05 level (Ducan’s test).

The independent lines (*sparr11-ko* line1, *sparr11-ko* line2, *spphl2-ko* line1, *spphl2-ko* line2, *spnf-ya10-ko* line1, *spnf-ya10-ko* line2, *spmyb84-ko* line1 and *spmyb84-ko* line2) were selected for rapid propagation, and identical mutations were confirmed in all clonal progenies. Subsequently, all of the chosen knockout lines were exposed to 1/4 MS solutions supplemented with Cd (100μM CdCl_2_) for ten days. The above-ground parts of the *sparr11-ko* and *spmyb84* mutants showed significantly reduced Cd concentrations to those of the wild-type plants after the 100 μM Cd treatment (Figure 5, Table S11). These outcomes suggest that the transcription factors SpARR11 and SpMYB84 may regulate the genes associated with Cd hyperaccumulation in *S. plumbizincicola*.

## 3. Discussion

*S. plumbizincicola* and *S. alfredii* (NHE) are closely related species belonging to the same genus. However, they exhibit contrasting abilities in cadmium accumulation capacity. *S. plumbizincicola*, a typical hyperaccumulator plant, has excellent biological properties to accumulate exceedingly high levels of Cd/Zn without exhibiting toxicity symptoms in its above-ground parts. This makes it greatly valuable for remediating Cd-contaminated soil. The molecular basis for hyperaccumulation in these plants lies in the high-abundance transcription and regulation of genes encoding heavy metal absorption, transport and detoxification. In this study, we utilized full-length transcriptomes of *S. plumbizincicola* as reference transcriptomes and combined them with next-generation sequencing to provide genetic information about *S. plumbizincicola* and *S. alfredii* (NHE). We also explored the differential expressions of structural genes and transcriptional regulatory genes that may be involved in Cd hyperaccumulation. Four differentially expressed transcription factor genes were chosen to create knockout mutants by using the CRISPR/Cas9 technique. Among these, two transcriptional regulatory genes, *SpARR11* and *SpMYB84*, were discovered to be relevant for Cd hyperaccumulation.

Compared to *S. alfredii* (NHE), *S. plumbizincicola* exhibited greater Cd uptake, root-to-shoot translocation and accumulation in shoots (Figure 1). This was reflected in the highly expressed genes related to metal transportation and detoxification associated with Cd hyperaccumulation and hypertolerance in *S. plumbizincicola* [9] (Table S7). For instance, *SpHMA2*, which encodes a product that is highly homologous to *Arabidopsis thaliana HMA2* and *Arabidopsis halleri HMA4*, was expressed in high abundance in *S. plumbizincicola* and may play a role in Cd transport from roots to shoot [5,21]. Similarly, some ZIP family proteins, such as ZIP1, ZIP4 and ZIP11, can transport cadmium. The expressions of these genes were found to be ten times higher in *S. plumbizincicola* roots than in *S. alfredii* (NHE) (Table S7). SaZIP4 has been identified as a Zn/Cd uptake transporter in *S. alfredii* Hance [22]. *AtZIP4*-knockout mutants also absorbed less Zn and Cd than the wild-type plants under Zn or Cd sufficient treatments, indicating that AtZIP4 is a Zn and Cd transporter in *A. thaliana* [22]. Metal chelation, sequestration in cell walls and vacuolar compartmentalization may be crucial mechanisms for metal detoxification in hyperaccumulators. For example, SpHMA3 has previously been shown to be crucial for Cd detoxification via vacuolar compartmentalization [10]. The expressions of *HMA3* in the young shoots of *S. plumbizincicola* were higher than those in *S. alfredii* (NHE) (Table S7). Metallothioneins (MTs), a class of cysteine-rich proteins, play important roles in chelating metals to detoxify heavy metal stress in plants [23]. SaMT2 and SpMT2 can significantly improve Cd tolerance and accumulation by chelating metals and improving antioxidant systems [24,25]. Sp-R_1-10k_transcript/46313 (*MT2*) gene was highly expressed in *S. plumbizincicola* (Table S7). These highly expressed genes involved in heavy metal transport, chelation and stress-related response may play a direct role in metal ion balance, heavy metal detoxification and hyperaccumulation in *S. plumbizincicola*.

Based on the high-abundance constitutive expression of metal transportation and detoxification genes, specific transcription factors are essential for regulating Cd hyperaccumulation in *S. plumbizincicola*. Differential expression analysis identified transcription factor genes from the AP2/ERF, MYB, bZIP, WRKY and GRAP gene families that were differentially expressed between *S. plumbizincicola* and *S. alfredii* (NHE) (Table S8). A few transcription factors that were constitutively expressed in *S. plumbizincicola* were induced by cadmium in *S. alfredii* (NHE) (Figure 2). The differential transcription factor genes between *S. plumbizincicola* and *S. alfredii* (NHE) were significantly enriched in plant hormone signal transduction pathways, suggesting that transcription factors may regulate the expression of hyperaccumulation-related genes by participating in hormone-signaling pathways (Figure 3). Certain transcriptional regulatory genes have been linked to cadmium stress in other plants. For instance, MYB43 negatively regulated cadmium tolerance in *Arabidopsis* by transcriptionally inhibiting the Cd transporters (HMA2, HMA3 and HMA4) [26]. MYB49 positively affected Cd accumulation in *A. thaliana* by directly regulating the expression of *bHLH38* and *bHLH101* and subsequently activating *IRT1* transcription [27]. Differentially expressed MYB genes were upregulated in *S. alfredii* (NHE) after cadmium induction, indicating that MYB transcription factors may be involved in the response to cadmium stress. In contrast, most of the MYB transcription factor genes were constitutionally expressed in *S. plumbizincicola*, further demonstrating that the genes related to cadmium hyperaccumulation were constitutionally expressed. After cadmium treatment, differentially expressed WRKY genes were upregulated in the root of *S. alfredii* (NHE) and the leaf of *S. plumbizincicola*, suggesting that WRKY genes might play a role in response to cadmium stress (Table S8). WRKY12 could bind directly to the W-box of GSH1 promoter in *A. thaliana* and indirectly inhibit the phytochelatin synthesis-related gene expression, thereby negatively regulating Cd accumulation and tolerance [28]. SaHsfA4c, a heat shock transcription factor, could enhance plant Cd tolerance by activating ROS-scavenging enzyme activity and upregulating the expression of *Hsps* in *S. alfredii* Hance [29]. Therefore, various transcription factors are involved in the response to Cd stress in plants. Among the large number of differentially expressed genes, constitutively and highly expressed transcription factors are likely candidates for regulation of cadmium hyperaccumulation in *S. plumbizincicola*.

**Figure A1:**
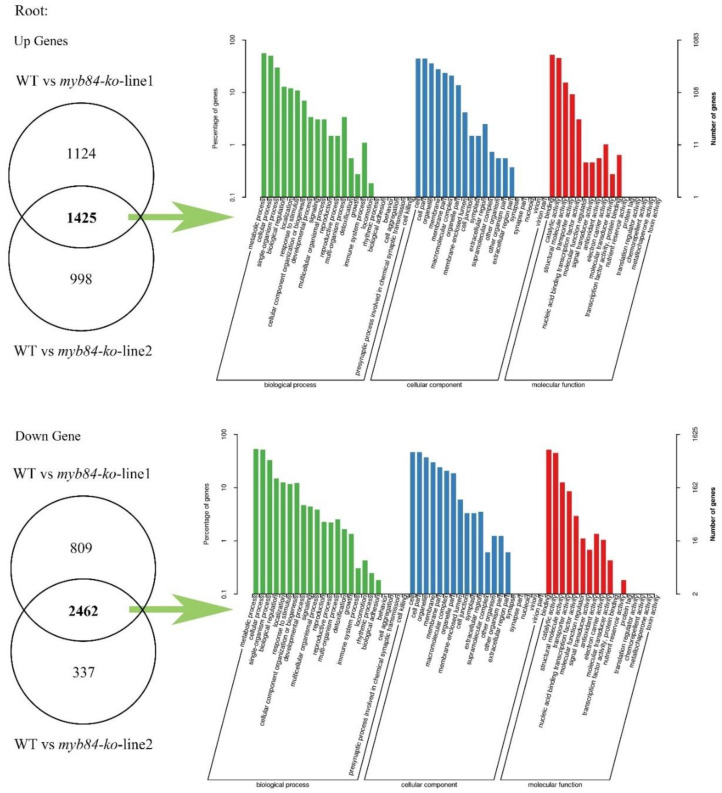
Differentially expressed gene analysis between SpMYB84 knockout strain and wild type. The Venn diagram shows the number of differentially expressed genes, and the bar chart shows the results of the GO analysis.

**Figure A2:**
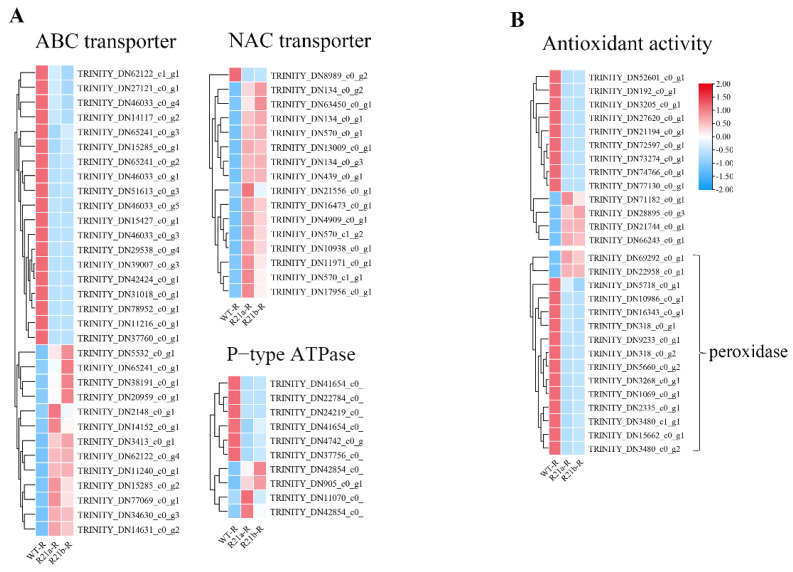
Differentially expressed gene heat maps of key enrichment pathways between *SpMYB84* knockout strain and wild type. Heat map of partial differential transporter (**A**). Heat map of differential genes in antioxidant activity pathways (**B**). Note: R1a: *sparr11-ko* line1; R1b: *sparr11-ko* line2; R21a: *spmyb84-ko* line1; R21b: *spmyb84-ko* line2.

**Figure A3:**
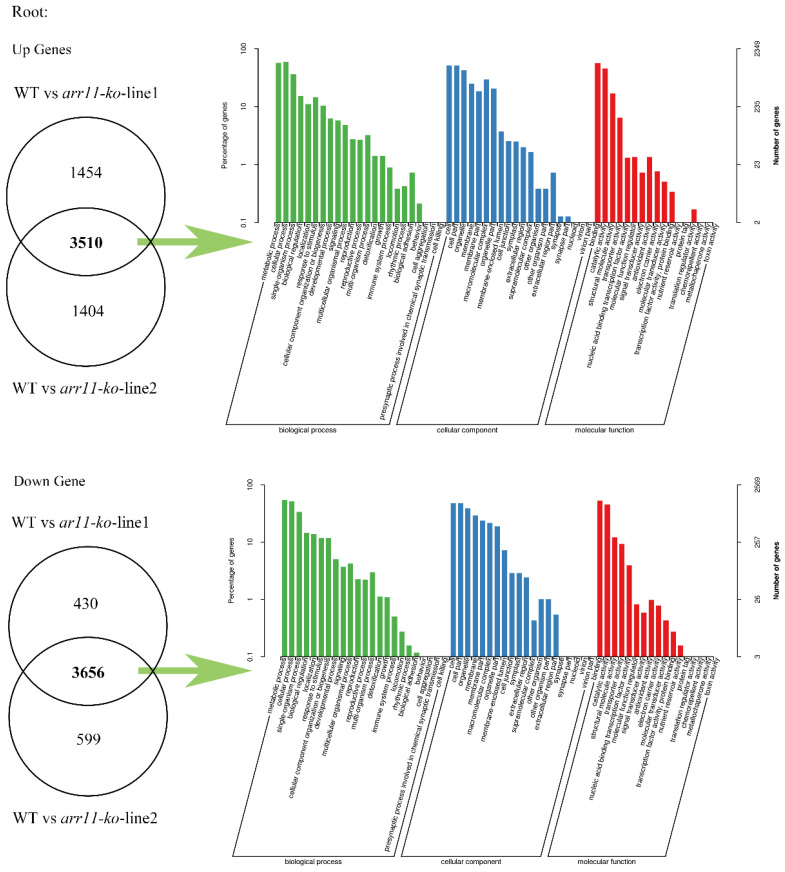
Differentially expressed gene analysis between *SpARR11* knockout strain and wild type. The Venn diagram shows the number of differentially expressed genes, and the bar chart shows the results of the GO analysis.

**Figure A4:**
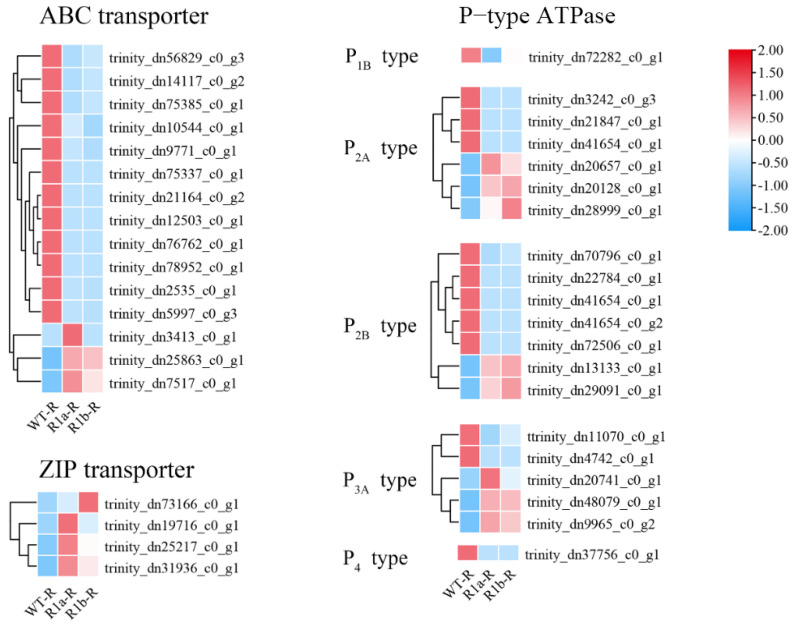
Heat map of partial differential transporter between *SpARR11* knockout strains and wild types. Note: R1a: *sparr11-ko* line1; R1b: *sparr11-ko* line2; R21a: *spmyb84-ko* line1; R21b: *spmyb84-ko* line2.

To investigate the potential role of candidate transcription factor genes in regulating Cd hyperaccumulation, we used CRISPR/Cas9 technology to create knockout mutations of transcription factors in *S. plumbizincicola*. Specifically, we selected four candidate transcription factor genes that were highly expressed in the roots of *S. plumbizincicola*. Our results indicated that the *sparr11* and *spmyb84* mutant lines exhibited decreased Cd accumulation in their above-ground parts compared to the wild-type plants (Figure 5). This suggests that SpMYB84 and SpARR11 may play a role in regulating the expression of Cd translocation-related genes in hyperaccumulator *S. plumbizincicola*. We, therefore, performed transcriptome sequencing of the mutants and wild-type plants, which revealed that the *SpMYB84* mutant may be involved in transcriptional regulation of transporter activity and antioxidant activity (Figure A1 and Figure A2). Specifically, many differentially expressed peroxidase-related genes in the antioxidant activity pathway were downregulated, while several differentially expressed genes in the transporter-activity pathway were annotated as ABC transporters, NAC transporters, P-type ATPase transporters, etc. (Figure A1 and Figure A2). In Arabidopsis, AtMYB84 has been shown to modulate phosphate accumulation in response to Zn deficiency [30]. Meanwhile, differential gene analysis in the roots indicated that the *SpARR11* mutant strains may also be involved in the transcriptional regulation of transporter activity, exhibiting significant differences in ABC transporter, ZIP transporter and P-type ATPase transporter (Figure A3 and Figure A4). Previous research has identified *AtARR11* as a novel regulator of SA/JA crosstalk involved in cytokinin-mediated responses [31]. Interestingly, the *sparr11-ko* knockout mutant exhibited distinct phenotypes with slower root growth and fewer adventitious roots than the wild type. While transcriptome sequencing yielded a wealth of gene expression data, further studies will be necessary to identify the targeted genes and cis-regulatory elements regulated by the transcription factors SpMYB84 and SpARR11 in *S. plumbizincicola*.

## 4. Materials and Methods

### 4.1. Plant Material and Growth Condition

For this study, two Sedum species with contrasting cadmium accumulation were utilized. Both the cadmium hyperaccumulator *S. plumbizincicola* and the non-accumulator *S. alfredii* (NHE) were collected from Chun’an and Fuyang counties, respectively, in Zhejiang Province, China [32]. Sedum plants were asexually propagated, and shoots measuring approximately 7 cm in height were selected for rooting in a hydroponic culture system in a greenhouse (Light: 16 h light/8 h dark; temperature: 23 °C). Plants with well-established roots and similar sizes were then chosen and treated in one-quarter Murashige and Skoog (MS) nutrient solution containing CdCl_2_ (0 or 50 μM) for a period of 10 days. Each experiment was biologically replicated four times for cadmium determination and three times for RNA-seq. Nutrient solutions were changed every three days to ensure optimal plant growth.

### 4.2. Determination of Cd Content in Plants

After the 10-day treatment period, the plants were harvested, and their roots were soaked in 10 mM EDTA in phosphate buffered saline (PBS) for 20 min to eliminate the cadmium ions that were adsorbed on the surface. The roots were then washed three times with the same PBS buffer. All of the harvested plants were divided into four parts (roots, stem, mature leaves and young leaves—a cluster of leaves at the shoot apex) and dried at 80 °C until a constant weight was obtained. The dried samples were nitrated in 1 mL HNO_3_ at 180 °C for 4 h. The concentrations of Cd were determined using an inductively coupled plasma optical emission spectrometer (iCAP 6300 ICP-OES; Thermo Electron Co., Waltham, MA, USA).

### 4.3. Transcriptome Sequencing

After the 10-day treatment period with or without Cd, samples of the roots and young shoots from the Sedum species were collected separately for RNA extraction and transcriptome sequencing (Allwegene, Beijing, China). Each experiment was biologically replicated three times with each individual plant used as one biological replicate. RNA purity and concentration were detected using a NanoDrop spectrophotometer (Thermo Scientific, Waltham, MA, USA), while RNA fragment length was detected using an Agilent Bioanalyzer 2100 (Agilent Technologies, Santa Clara, CA, USA). A total amount of 1.5 μg RNA per sample was utilized for cDNA library construction for each sample. Twenty-four sequencing libraries were generated using the NEBNext^®^ Ultra™ RNA Library Prep Kit for Illumina^®^ (NEB, Ipswich, MA, USA) by Allwegene (Beijing, China). The *S. plumbizincicola* and *S. alfredii* (NHE) transcriptome sequencing data have been uploaded to the NCBI database with the entry number PRJNA932212. *S. plumbizincicola* full-length transcriptome data can be found under PRJNA934668, which includes RNA from the roots, stems and leaves. Short, clean sequence reads from each RNA-seq library were aligned to the *S. plumbizincicola* full-length reference transcriptome to obtain unique mapped reads using RSEM software (v1.2.15) [33].

### 4.4. Transcriptome Analysis

To obtain high-quality clean data for downstream analyses, raw reads were screened to exclude those with sequencing adapters, low quantity reads and reads containing ploy-N. The remaining clean reads were then mapped to the reference full-length transcriptome sequence using STAR. Differential expression analysis was conducted using the DESeq R package (1.10.1), and genes with FDR < 0.05 and |log2FC| > 1 were considered differentially expressed. Gene ontology (GO) enrichment analysis of the differentially expressed genes was performed through the GOseq R package [34]. In vivo metabolic analysis was carried out using the KEGG database (Kyoto Encyclopedia of Genes and Genomes, http://www.kegg.jp), accessed on 20 September 2022.

Eight differentially expressed genes were selected for validation through quantitative real-time PCR (qRT-PCR). The cDNA were reverse transcribed using FastKing gDNA Dispelling RT SuperMix kit (Tiangen, Beijing, China). The primer pairs were designed by Primer Premier 5 and are listed in supplemental Table S12. The qRT-PCR reaction was carried out using 2× HQ SYBR qPCR Mix (Zoman, Beijing, China). Each sample was run in triplicate on a fluorescence quantitative instrument (qTOWER3Gtouch, Germany, Analytik Jena). The relative expression of genes was quantified using the 2^−ΔΔCT^ method.

### 4.5. Transmembrane, Subcellular and Motif Prediction of Hyperaccumulation-Related Candidate Genes

To predict transmembrane helices, we used the TMHMM-2.0 algorithm (https://services.healthtech.dtu.dk/service.php?TMHMM-2.0 (accessed on 20 July 2023)). Subcellular localizations were predicted using the Plant-mSubP tool (http://bioinfo.usu.edu/Plant-mSubP/ (accessed on 20 July 2023)). Transcription factor motif prediction was performed using MOTIF Search (https://www.genome.jp/tools/motif/ (accessed on 20 July 2023)).

### 4.6. Transcription Factor Genes Cloning and Knockout Vector Construction

We selected four constitutively expressed transcription factors that were highly expressed in *S. plumbizincicola* for cloning from the differentially expressed transcription factors between *S. plumbizincicola* and *S. alfredii* (NHE) in the root. Genomic fragments of these four genes were amplified based on the transcriptome sequence. Primers were designed using Primer Premier 5.0 and are listed in supplemental Table S1. We predicted the knockout target sites and designed corresponding knockout primers (Table S2) according to the method of Xing et al. [35] after sequencing and comparing with the transcriptome. PCR fragments were then amplified from pCBC-DT1T2 using the four primers and inserted between the two *Bsa* I sites of the binary vector pHSE401 by Golden Gate cloning; then, we obtained four knockout vectors after sequencing them [35,36].

### 4.7. Plant Transformations

To grow and transform *S. plumbizincicola*, we followed the method of Liu et al. [10]. Stem segments of *S. plumbizincicola* were used to induce high-frequency cluster buds, which were then used as explants for *Agrobacterium*-mediated genetic transformation. *Agrobacterium tumefaciens* carrying the transcription factor knockout vector was genetically transformed into *S. plumbizincicola*. After differentiation and hygromycin screening that lasted for more than three months, the shoots were induced to root on phytohormone-free MS medium containing hygromycin. Genomic DNA was extracted from the transformed seedlings, and the genomic region around the CRISPR target sites was amplified with PCR. The CRISPR/Cas9 transgenic lines (knockout mutants) targeting the transcription factor genes were obtained after sequencing identification.

### 4.8. Determination of Cadmium Content in Transgenic Knockout Plants

Shoot branches of similar sizes from both wild-type and transgenic plants were cut and rooted in a one-quarter MS nutrient solution for two weeks. Well-grown plants were then treated with or without 100 μM CdCl_2_ for 10 days. For determination of Cd content, each sample was biologically replicated three times with each biological replicate consisting of corresponding tissue from an individual plant. Refer to Section 4.2 for details on content determination.

### 4.9. Statistical Analysis

For the analysis of data from multiple groups, we used one-way analysis of variance (ANOVA) with Duncan’s multiple range test, and differences were considered significant at the 0.05 level. For the analysis of data from two groups, we used unpaired *t* tests. All analyses were performed using IBM SPSS STATISTICS 21.

## 5. Conclusions

In summary, our study demonstrated that the two Sedum species exhibit different characteristics of Cd accumulation. By analyzing transcriptome data from the second- and third-generation sequencing, we identified several heavy metal transport/detoxification-related genes, and transcription factor genes were highly and constitutively expressed in hyperaccumulator *S. plumbizincicola*. Using the CRISPR/Cas9 system, we knocked out four candidate transcription factor genes in *S. plumbizincicola* and observed that two of them may play a role in regulating cadmium hyperaccumulation, as they exhibited lower Cd accumulation in the above-ground parts of the mutant lines. In the future, integrating transcriptome analysis with CRISPR/Cas9 technology can help identify more Cd hyperaccumulation-related transcription factors by generating specific knockout mutants. Further identification and functional verification of their targeted genes will enhance our understanding of the transcriptional regulation mechanism of hyperaccumulation in *S. plumbizincicola*.

## Figures and Tables

**Table 1:** The list of 15 transcription factors’ DEGs highly expressed in *Sedum plumbizincicola*.

Gene ID	Best-Hit-A.th ^1^	Gene Name	Gene Family	Motif ^2^	Sa0YS ^3^	Sa0R ^3^	Sp0YS ^3^	Sp0R ^3^
Sp-R_1-10k_transcript/44752	AT2G41130.1	BHLH106	bHLH	HLH	6.83	25.97	10.49	77.64
Sp-R_1-10k_transcript/39703	AT4G02590.2	BHLH059	bHLH	HLH, Tom22	6.36	13.10	14.80	42.77
Sp-R_1-10k_transcript/42007	AT1G42990.1	BZIP60	bZIP	bZIP_1, bZIP_2,	2.56	7.45	8.61	25.85
Sp-R_1-10k_transcript/40522	AT1G75390.1	BZIP44	bZIP	bZIP_1, bZIP_2,	0.69	3.04	23.96	82.14
Sp-R_1-10k_transcript/35867	AT3G16857.2	ARR11	GARP	Response_reg, Myb_DNA-binding,	0.17	0.63	0.63	23.22
Sp-R_1-10k_transcript/39786	AT4G13640.1	PHL3	GARP	Myb_CC_LHEQLE, Myb_DNA-binding,	5.44	17.36	17.51	76.14
Sp-R_1-10k_transcript/38907	AT3G24120.1	PHL2	GARP	Myb_CC_LHEQLE, Myb_DNA-binding,	4.68	6.82	13.02	23.05
Sp-R_1-10k_transcript/17174	AT1G14350.1	MYB124	MYB	Myb_DNA-binding, Myb_DNA-bind_6,	4.05	5.51	14.93	13.85
Sp-R_1-10k_transcript/38968	AT3G49690.1	MYB84	MYB	Myb_DNA-binding, Myb_DNA-bind_6	0.05	11.72	0.15	64.96
Sp-R_1-10k_transcript/39578	AT5G57620.1	MYB36	MYB	Myb_DNA-binding, Myb_DNA-bind_6,	0.15	8.69	0.19	37.67
Sp-R_1-10k_transcript/38848	AT5G06510.1	NF-YA10	NF-Y	CBFB_NFYA	1.10	3.14	2.39	23.14
Sp-R_1-10k_transcript/40870	AT1G29280.1	WRKY65	WRKY	WRKY	4.50	12.28	2.51	37.59
Sp-R_1-10k_transcript/21995	AT2G37260.1	WRKY44	WRKY	WRKY, FLYWCH	0.61	4.15	1.59	62.10
Sp-R_1-10k_transcript/26282	AT2G30590.1	WRKY21	WRKY	WRKY, Plant_zn_clust	9.34	13.06	9.79	35.90
Sp-R_1-10k_transcript/41387	AT2G23320.1	WRKY15	WRKY	WRKY, Plant_zn_clust	4.89	7.71	21.96	90.26

^1^ Best-Hit-A.th refers to the Arabidopsis thaliana gene that is most closely homologous to the transcript ID of *Sedum plumbizincicola*. ^2.^ For each gene, one or two motifs was observed, while additional information can be found in Table S10. ^3.^ FPKM(Sp0R) > 20 or FPKM(Sp50R) > 20 or FPKM(Sp0YS) > 20 or FPKM(Sp50YS) > 20; log2(Sp0R/Sa0R) > 1 or log2(Sp0YS/Sa0YS) > 1. Here, the mean value of FPKM was used to represent the expression level of each sample. Table S10 contains comprehensive data on 265 transcription factor genes.

## Data Availability

The RNA-Seq was uploaded to the National Center for Biotechnology Information with number PRJNA932212. The Gene and protein sequences of *SpARR11*, *SpPHL2*, *SpNF-YA10* and *SpMYB84* have been uploaded to GenBank, and the submission ID was 2696843, 2696894, 2696955, 2696962. Other data are included in this published article and its Supplementary Information Files.

## Supplementary Materials

The following supporting information can be downloaded at: https://www.mdpi.com/article/10.3390/ijms241411845/s1.

## Appendix A

### Appendix A.1. Transcriptome Analysis of Knockout Mutants from SpARR11 and SpMYB84

#### Appendix A.1.1. Transcriptome Sequencing

The mutant *sparr11-ko* line1, *sparr11-ko* line2, *spmyb84*-ko line1, *spmyb84*-ko line2 and wild-type *S. plumbizincicola* were selected as materials with no biological duplication. After culturing for 10 days in one-quarter MS nutrient solution, the roots were respectively taken and placed in liquid nitrogen for transcriptome sequencing (BioMarker, Beijing, China).

#### Appendix A.1.2. Transcriptome Analysis

The mutant transcriptome was analyzed without reference. Log2(Fold Change) ≥ 2 and FDR < 0.01 were used as the screening criteria for differentially expressed genes.

#### Appendix A.1.3. Genes Knockout Mutants Transcriptome Differential Gene Analysis

GO analysis was performed on the common differentially expressed genes (wild type and *spmyb84-ko line1* vs. wild type and *spmyb84-ko line2*). The differentially expressed genes in the root using the molecular functional module showed significant differences in “transporter activity” and “antioxidant activity”. The differential genes of the antioxidant activity pathway were extracted, and most peroxidase-related genes were downregulated. There were significant differences in the “transporter activity” pathway, some of which were annotated as “metal ion transport” with more downregulation. In addition, MapMan was used to obtain the differential transporter genes. Through analysis, it was found that ABC transporter, NAC transporter and P-type ATPase transporter were significantly different between the wild type and *myb84-ko*.

GO analysis was performed on the common differentially expressed genes (wild type and *sparr11-ko line1* vs. wild type and *sparr11-ko line2*). In the molecular functional module, the “transporter activity” was significantly different. MapMan was used to identify the modulated differentially expressed transporter genes, and it was found that ABC transporter, ZIP transporter and P-type ATPase transporter were significantly different between wild type and *sparr11-ko*.
