# Supplementary material for: Exploring Transcriptional Regulation of Hyperaccumulation in Sedum plumbizincicola through Integrated Transcriptome Analysis and CRISPR/Cas9 Technology
Source: Int J Mol Sci. 2023 Jul 24;24(14):11845. doi: 10.3390/ijms241411845 (PMC10380820; doi:10.3390/ijms241411845)
Supplement: Supplementary file 1 [file ijms-24-11845-s001.zip › Figure S3.pdf]

(a)

```
                               *        20         *        40         *        60         *        80
Wild-type SpARR11        : MMDTTTRDRTGRVVVDDDTWKKMKKCYVTTCCARDARRGRKDGDVSDVNMDMGKGMDVNNASKKRKDVADKCSTTSDTG :  80
sparr11-ko line1 allele 1 : MMDTTTRDRTGRVVVDDDTWKKMKKCYVTTCCARDARRGRKDGDVSDVNMDMGKGMDVNNASKKRKDVADKCSTTSDTG :  80
sparr11-ko line1 allele 2 : MMDTTTRDRTGRVVVDDDTWKKMKKCYVTTCCARDARRGRKDGDVSDVNMDMGKGMDVNNASKKRKDVADKCSTTSDTG :  80
sparr11-ko line2 allele 1 : MMDTTTRDRTGRVVVDDDTWKKMKKCYVTTCCARDARRGRKDGDVSDVNMDMGKGMDVNNASKKRKDVADKCSTTSDTG :  80
sparr11-ko line2 allele 2 : MMDTTTRDRTGRVVVDDDTWKKMKKCYVTTCCARDARRGRKDGDVSDVNMDMGKGMDVNNASKKRKDVADKCSTTSDTG :  80

                               *       100         *       120         *       140         *       160
Wild-type SpARR11        : STSKRARVVWTVDHKKAVKGKGKKDSMNVWTRNVASHKYRYSRKDNNGDKRVSDHDVDCKKNSDHTNDDSRNRKGDKSDA : 160
sparr11-ko line1 allele 1 : STSKRARVVWTVDHKKAVKGKGKKDSMNVWTRNVASHKYRYSRKDNNGDKRVSDHDVDCKKNSDHTNDDSRNRKGDKSDA : 160
sparr11-ko line1 allele 2 : STSKRARVVWTVDHKKAVKGKGKKDSMNVWTRNVASHKYRYSRKDNNGDKRVSDHDVDCKKNSDHTNDDSRNRKGDKSDA : 160
sparr11-ko line2 allele 1 : STSKRARVVWTVDHKKAVKGKGKKDSMNVWTRNVASHKYRYSRKDNNGDKRVSDHDVDCKKNSDHTNDDSRNRKGDKSDA : 160
sparr11-ko line2 allele 2 : STSKRARVVWTVDHKKAVKGKGKKDSMNVWTRNVASHKYRYSRKDNNGDKRVSDHDVDCKKNSDHTNDDSRNRKGDKSDA : 160

                               *       180         *       200         *       220         *       240
Wild-type SpARR11        : DKCDSDAKVTVARHMSDHSADDNNRKYWNDSNNSHHYYRSTRNNCSRADSAVAGKDYKRWDNHGRDADDMGSSHDARDWC : 240
sparr11-ko line1 allele 1 : DKCDSDAKVTVARHMSDHSAGRG*-------------------------------------------------------- : 183
sparr11-ko line1 allele 2 : DKCDSDAKVTVARHMSDHSADDNNRKYWNDSNNSHHYYRSTRNNCSRADSAVAGKDYKRWDNHG---TRM*--------- : 227
sparr11-ko line2 allele 1 : DKCDSDAKVTVARHMSDHSAGRG*-------------------------------------------------------- : 183
sparr11-ko line2 allele 2 : DKCDSDAKVTVARHMSDHSADDNNRKYWNDSNNSHHYYRST-NNCSRADSAVAGKDYKRWDNHG---TRM*--------- : 226

                               *       260         *       280         *       300         *
Wild-type SpARR11        : GASCDTAHSSSYYVKSDSDCNDGMKCTDGKKVACRTRSSGGDRTARSGDGRGRARVTDGVARHRDRKAWARM* : 312
sparr11-ko line1 allele 1 : ------------------------------------------------------------------------- :  -
sparr11-ko line1 allele 2 : ------------------------------------------------------------------------- :  -
sparr11-ko line2 allele 1 : ------------------------------------------------------------------------- :  -
sparr11-ko line2 allele 2 : ------------------------------------------------------------------------- :  -
```

(b)

```
                             *        20         *        40         *        60         *        80
Wild-type SpPHL2        : MYSAHSGDGSVYHRHSSSDCTNGDSCVMTSDKRRWTAHDRVDAVAGGDKATKTRTMGVKGTYHKSHKYRGKAGKSTNSKS :  80
spphl2-ko line1 allele 1 : MYSAHSGDGSVYHRHSSSDCTNGDSCVMTSDKRRWTAHDRVDAVAGGDKATKTRTMGVKGTYHKSHKYRGKAGKSTNSKS :  80
spphl2-ko line1 allele 2 : MYSAHSGDGSVYHRHSSSDCTNGDSCVMTSDKRRWTAHDRVDAVAGGDKATKTRTMGVKGTYHKSHKYRGKAGKSTNSKS :  80
spphl2-ko line2 allele 1 : MYSAHSGDGSVYHRHSSSDCTNGDSCVMTSDKRRWTAHDRVDAVAGGDKATKTRTMGVKGTYHKSHKYRGKAGKSTNSKS :  80
spphl2-ko line2 allele 2 : MYSAHSGDGSVYHRHSSSDCTNGDSCVMTSDKRRWTAHDRVDAVAGGDKATKTRTMGVKGTYHKSHKYRGKAGKSTNSKS :  80

                             *       100         *       120         *       140         *       160
Wild-type SpPHL2        : VRKRSSYVTARVMVRKHVRRRSGKYRKACKANDTVAAAGAARSAKSHDSSDKMSSDTTTDSSRKTNVVRGDCSVNSCTST : 160
spphl2-ko line1 allele 1 : VRKRSSYVTARVMVRKHVRRRSGKYRKACKANDTVAAAGAARSAKSHDSSDKMSSDTTTDSSRKT-TVRGDCSVNSCTST : 159
spphl2-ko line1 allele 2 : VRKRSSYVTARVMVRKHVRRRSGKYRKACKANDTVAAAGAARSAKSHDSSDKMSSDTTTDSSRKT-TVRGDCSVNSCTST : 159
spphl2-ko line2 allele 1 : VRKRSSYVTARVMVRKHVRRRSGKYRKACKANDTVAAAGAARSAKSHDSSDKMSSDTTTDSSRKTTCDR-HCSVCTCTA- : 158
spphl2-ko line2 allele 2 : VRKRSSYVTARVMVRKHVRRRSGKYRKACKANDTVAAAGAARSAKSHDSSDKMSSDTTTDSSRKT----------TC*-- : 147

                             *       180         *
Wild-type SpPHL2        : ATHGSASNANAAKKRRVGNSSNMAVWMHSG* : 190
spphl2-ko line1 allele 1 : ATHGSASNANAAKKRRVGNSSNMAVWMHSG* : 189
spphl2-ko line1 allele 2 : ATHGSASNANAAKKRRVGNSSNMAVWMHSG* : 189
spphl2-ko line2 allele 1 : --WGSSK*----------------------- : 163
spphl2-ko line2 allele 2 : ------------------------------- :  -
```

(c)

```
                               *        20         *        40         *        60         *        80
Wild-type SpNF-YA10        : MTTVYKYTNTGHSSATMRWTAGSHSTASKSVNTTKTDDSMVKDVKNRVANTSSGTRTVRASVTVKTSYHSDSGGSMVYTY :  80
spnf-ya10-ko line1 allele 1 : MTTVYKYTNTGHSSATMRWTAGSHSTASKSVNTTKTDDSMVKDVKNRVANTSSGTRTVRASVTVKTSYHSDSGGSMVYTY :  80
spnf-ya10-ko line1 allele 2 : MTTVYKYTNTGHSSATMRWTAGSHSTASKSVNTTKTDDSMVKDVKNRVANTSSGTRTVRASVTVKTSYHSDSGGSMVYTY :  80
spnf-ya10-ko line2 allele 1 : MTTVYKYTNTGHSSATMRWTAGSHSTASKSVNTTKTDDSMVKDVKNRVANTSSGTRTVRASVTVKTSYHSDSGGSMVYTY :  80
spnf-ya10-ko line2 allele 2 : MTTVYKYTNTGHSSATMRWTAGSHSTASKSVNTTKTDDSMVKDVKNRVANTSSGTRTVRASVTVKTSYHSDSGGSMVYTY :  80

                               *       100         *       120         *       140         *       160
Wild-type SpNF-YA10        : SYGDSYGVMSAYGMAGRMMDSSDDGYVNAKYNGRRRHRAKAVSRTKARKAYMHSRHHARRRGCGGRKTKTDGGMSNSNSM : 160
spnf-ya10-ko line1 allele 1 : SYGDSYGVMSAYGMAGRMMDSSDDGYVNAKYNGRRRHRAGGTS-NHSSGVYA*--------------------------- : 131
spnf-ya10-ko line1 allele 2 : SYGDSYGVMSAYGMAGRMMDSSDDGYVNAKYNGRRRHRARRN*------------------------------------- : 122
spnf-ya10-ko line2 allele 1 : SYGDSYGVMSAYGMAGRMMDSSDDGYVNAKYNGRRRHRAGGTS-NHSSGVYA*--------------------------- : 131
spnf-ya10-ko line2 allele 2 : SYGDSYGVMSAYGMAGRMMDSSDDGYVNAKYNGRRRHRARGTNGNNSRGYMAGSRRGT---GCGGRKTKTDGGMSNSNSM : 157

                               *       180         *       200         *
Wild-type SpNF-YA10        : TSHSSSGSDNVTNSKTHTGNSGSATSMYTRGNCCNANTARGVAHCCCNKA* : 210
spnf-ya10-ko line1 allele 1 : --------------------------------------------------- :  -
spnf-ya10-ko line1 allele 2 : --------------------------------------------------- :  -
spnf-ya10-ko line2 allele 1 : --------------------------------------------------- :  -
spnf-ya10-ko line2 allele 2 : TSHSSSGSDNVTNSKTHTGNSGSATSMYTRGNCCNANTARGVAHCCCNKA* : 207
```

(d)

```
                                *        20         *        40         *        60         *        80
Wild-type SpMYB84  allele 1 : MGRACCDKANVKKGWSDTKKSYNGTGGNWAKGKRCGKSCRRWNYRNRHGGSDNCSYSGSRWSAAGRTDNDKNYWNTRKKK :  80
Wild-type SpMYB84  allele 2 : MGRACCDKANVKKGWSDAKKSYNGTGGNWAKGKRCGKSCRRWNYRNRHGGSDNCSYSGSRWSAAGRTDNDKNYWNTRKKK :  80
spmyb84-ko line1 allele 1   : MGRACCDKANVKKGWSDTKKSYNGTGGNWAKGKRCGKSCRRWNYRNRHGGSDNCSYSGSRWSAAGRTDNDKNYWNTRKKK :  80
spmyb84-ko line1 allele 2   : MGRACCDKANVKKGWSDAKKSYNGTGGNWAKGKRCGKSCRRWNYRNRHGGSDNCSYSGSRWSAAGRTDNDKNYWNTRKKK :  80
spmyb84-ko line2 allele 1   : MGRACCDKANVKKGWSDTKKSYNGTGGNWAKGKRCGKSCRRWNYRNRHGGSDNCSYSGSRWSAAGRTDNDKNYWNTRKKK :  80
spmyb84-ko line2 allele 2   : MGRACCDKANVKKGWSDAKKSYNGTGGNWAKGKRCGKSCRRWNYRNRHGGSDNCSYSGSRWSAAGRTDNDKNYWNTRKKK :  80
```

```
                                          *        100         *        120         *        140         *       160
Wild-type SpMYB84  allele 1  : GKRKHYNARRSGGNMMTMNKVSYWVSYSNDARGVNDHTSRRMKGGRSASDHSNGSMVSNNVMSSNMYSGSTGGYVSSSDD : 160
Wild-type SpMYB84  allele 2  : GKRKHYNARRSGWNMMTMNKVSYWVSYSNDARGVNDHTSRRMKGGRSTSD-SNGSMVSNNVMSSNMYSGSTGGYVSSSDD : 159
spmyb84-ko line1 allele 1    : GKRKHYNARRSGGNMMTMNKVSYWVSYSNDARGVNDHTSRRMKGGRSASDHSNGSMVSNNVMSSNMYSGSTGGYVSSSDD : 160
spmyb84-ko line1 allele 2    : GKRKHYNARRSGWNMMTMNKVSYWVSYSNDARGVNDHTSRRMKGGRSTS--SNGSMVSNNVMSSNMYSGSTGGYVSSSDD : 158
spmyb84-ko line2 allele 1    : GKRKHYNARRSGGNMMTMNKVSYWVSYSNDARGVNDHTSRRMKGGRSASD--HSSMVSNNVMSSNMYSGSTGGYVSSSDD : 158
spmyb84-ko line2 allele 2    : GKRKHYNARRSGWNMMTMNKVSYWVSYSNDARGVNDHTSRRMKGGRSTSD-SNGSMVSNNVMSSNMYSGSTGGYVSSSDD : 159

                                          *        180         *        200         *        220         *
Wild-type SpMYB84  allele 1  : THNNTYHSSGSGMDMGSHGGGNSSSNGMVDGNSTDDYNNNNNRNVDSNMTVDMARSSSVYNHYSVHSTTTNVNKYDH* : 237
Wild-type SpMYB84  allele 2  : THNNTYHSSGSGMDMGSYGGGNSSSHGMVDGNSTDDYNNN--RNVDSNMTVDMARSSSVYNHYSVHSTTTNVNKYDH* : 234
spmyb84-ko line1 allele 1    : THNNTYHDCWCYKDHSKSRGS*-------------------------------------------------------- : 181
spmyb84-ko line1 allele 2    : THNNTYHDCWCYKDHSKSRGS*-------------------------------------------------------- : 179
spmyb84-ko line2 allele 1    : THNNTYH--------------------------------------------------WVRNGY-VTRT*--------- : 175
spmyb84-ko line2 allele 2    : THNNTYHSSGTGMDMGSYGGGNSSSHGMVDGNSTDDYNNN--RNVDSNMTVDMARSSSVYNHYSVHSTTTNVNKYDH* : 234
```
