# Supplementary figures and images for: Exploring Transcriptional Regulation of Hyperaccumulation in Sedum plumbizincicola through Integrated Transcriptome Analysis and CRISPR/Cas9 Technology
Source: Int J Mol Sci. 2023 Jul 24;24(14):11845. doi: 10.3390/ijms241411845 (PMC10380820; doi:10.3390/ijms241411845)

# Correlation Heatmap

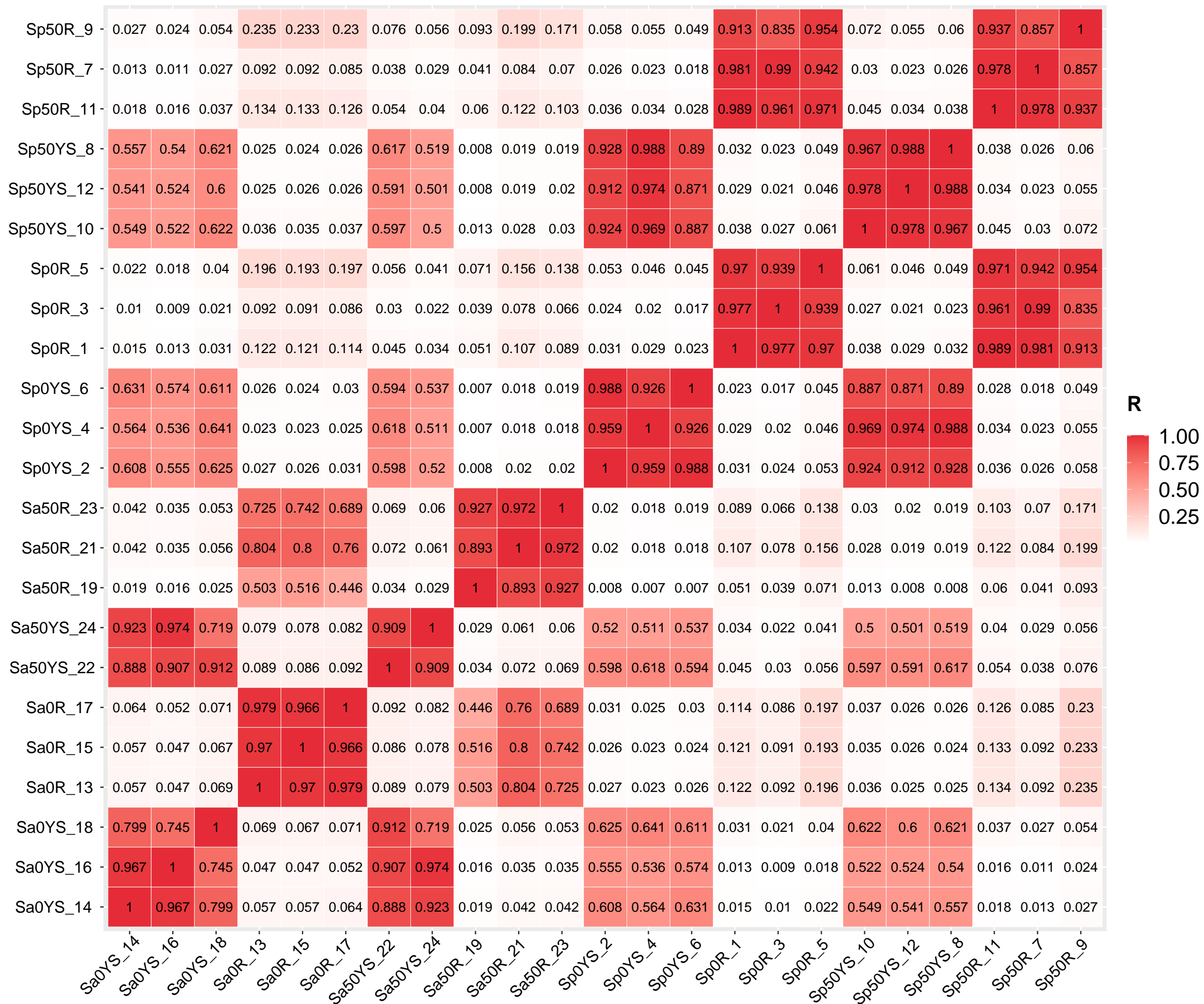

Supplement: Supplementary file 1 [file ijms-24-11845-s001.zip › Figure S1.pdf]

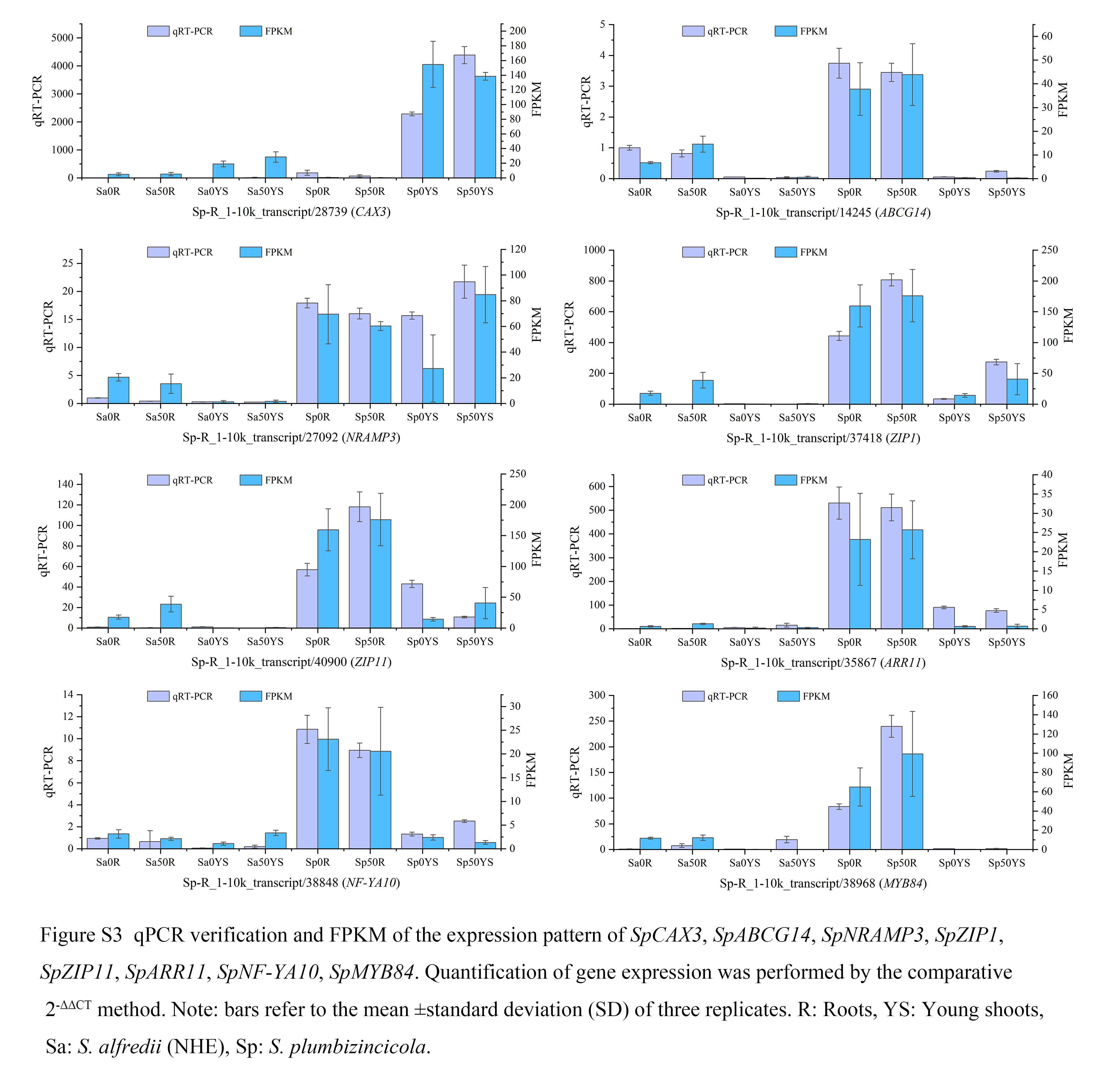

Supplement: Supplementary file 1 [file ijms-24-11845-s001.zip › Figure S2.tif]

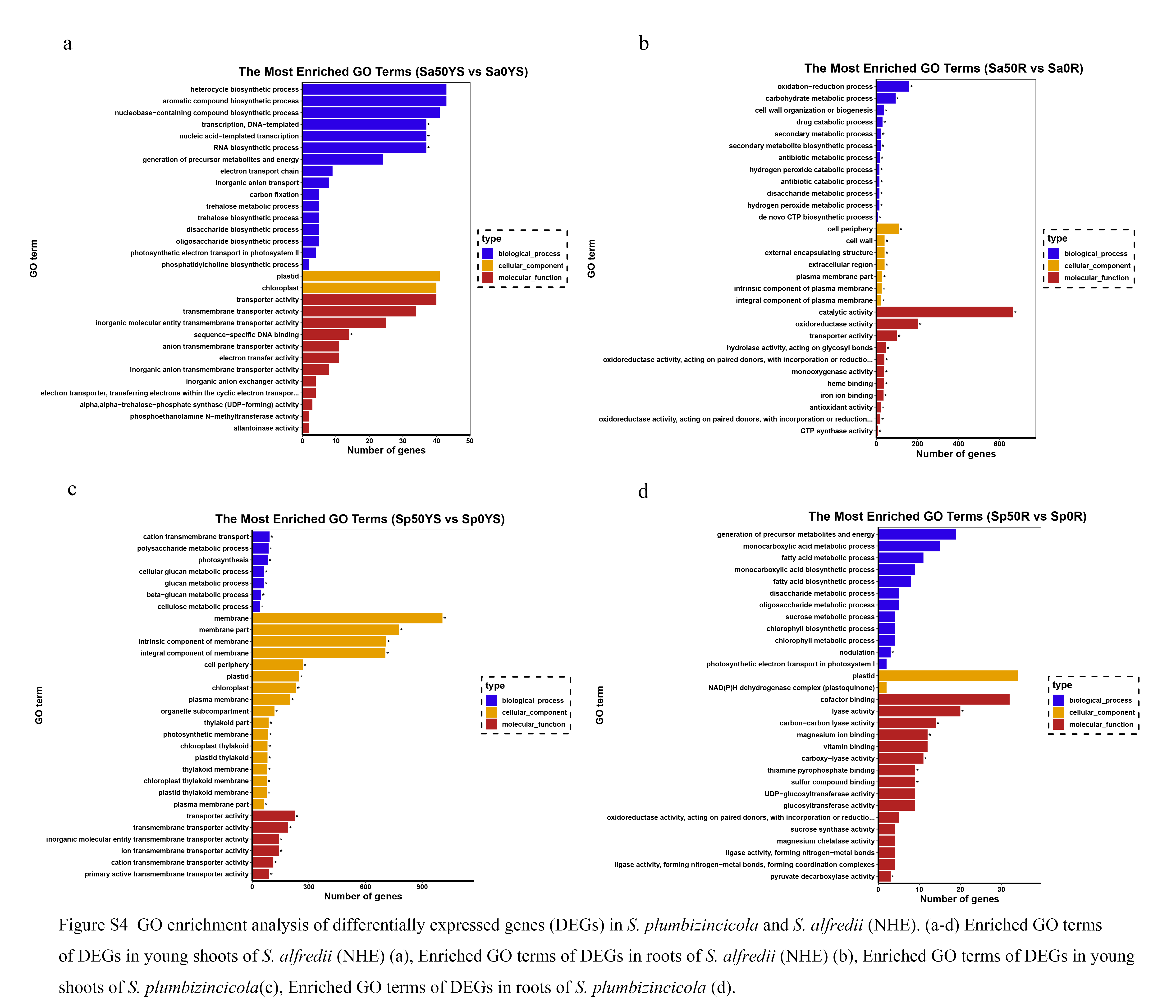

Supplement: Supplementary file 1 [file ijms-24-11845-s001.zip › Figure S4.tif]

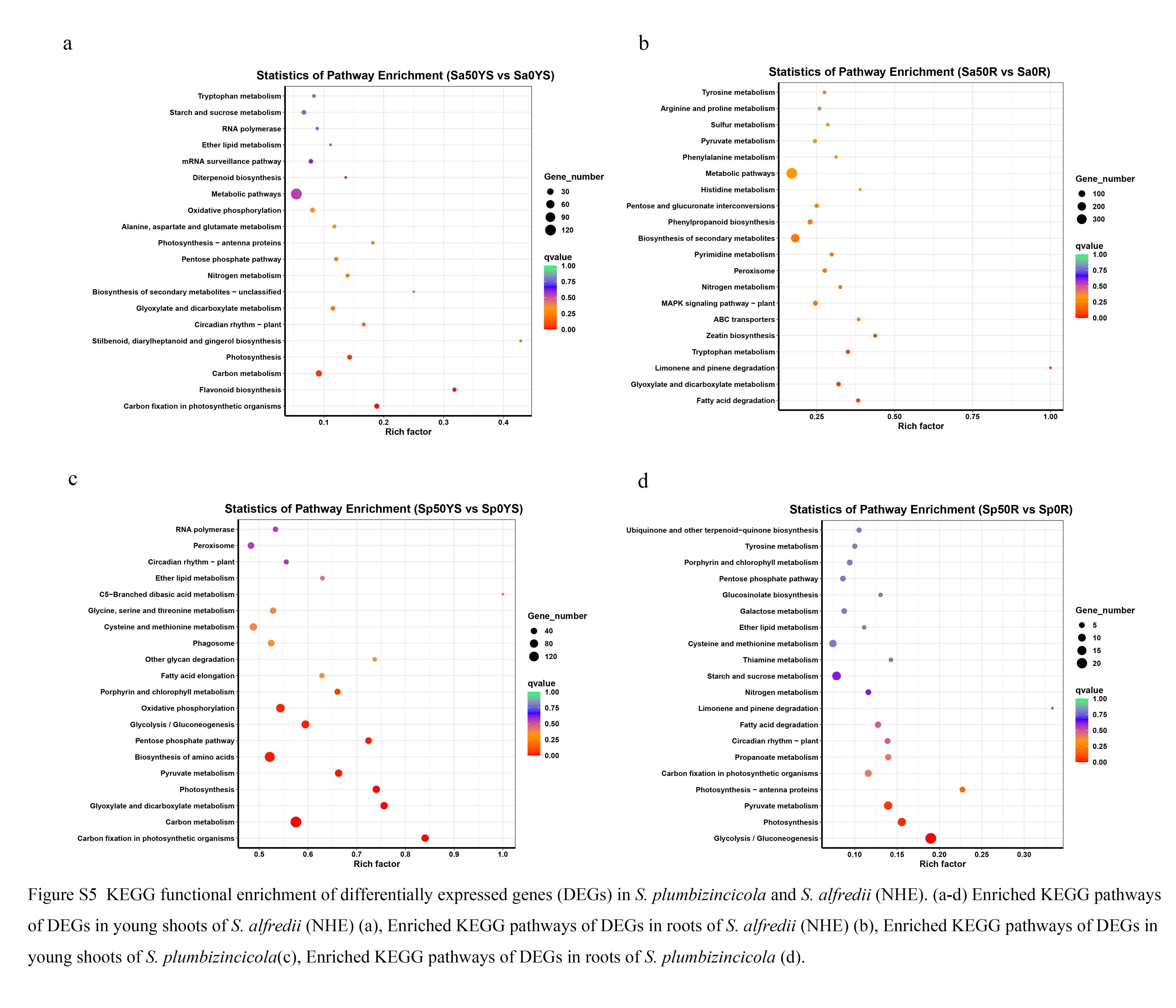

Supplement: Supplementary file 1 [file ijms-24-11845-s001.zip › Figure S5.tif]
